# Household clean energy consumption and health: Theoretical and empirical analysis

**DOI:** 10.3389/fpubh.2022.945846

**Published:** 2022-09-13

**Authors:** Fanghua Li, Wei Liang, Abbas Ali Chandio, Dungang Zang, Yinying Duan

**Affiliations:** ^1^College of Economics, Sichuan Agricultural University, Chengdu, China; ^2^School of Business and Tourism, Sichuan Agricultural University, Chengdu, China

**Keywords:** clean energy consumption, health, theory of health economics, CHARLS, China, ordered probit model

## Abstract

The impact of energy consumption on health has become a widely debated topic around the world. However, much of the current research on this topic lacks a theoretical basis. As a result, this paper employs both theoretical and empirical analysis to investigate the impact of household clean energy consumption on residents' health. First, based on the theories of health economics and energy economics, this paper believes that the usage of clean energy can improve the health of residents. Then, the sample for this study is comprised of data from the 2018 China Health and Retirement Longitudinal Study, and the Order Probit Model is applied for the empirical analysis. The outcomes of basic regression, robustness testing, and the treatment of endogenous factors reveal that the usage of clean energy has greatly benefited the health of residents. Furthermore, the heterogeneity analysis shows that long-term use of clean energy greatly improved the health of non-religious people and had a more pronounced impact on the health of women and low-income residents. In addition, the mechanistic analysis indicates that subjective happiness and air quality played a partial mediating role in the impact of cleaner energy consumption on health. Finally, cleaner household energy reduced the prevalence of hypertension, hyperlipidemia, lung disease, asthma, and depression. The conclusion of this paper supports the view of some existing literature, and several policy recommendations are made based on the research findings.

## Introduction

The health crisis is an obstacle to the sustainable development of individuals, families, nations, and the world (1, 2). From 2000 to 2020, the global mortality rate due to several diseases showed a continuous increasing trend (excluding deaths due to SARS and COVID-19) (3). All inhabitants of the world are threatened by various diseases, but health problems are more serious in developing countries. In the past 10 years, the number of deaths in China has increased by about 10 million each year (excluding deaths from COVID-19) (4). Many studies discussed the influencing factors of health from different perspectives, and some studies investigated the impact of household clean energy consumption on individual health.

Twumasi et al. (5) used the Order Probit Model to analyze research data from Ghana, and the results showed that the use of clean cooking fuels increased the proportion of healthy household members by 19.11%. Cleaner household energy improves indoor air quality (6) and reduces the probability of residents being diagnosed with respiratory diseases such as asthma, bronchitis, tuberculosis, and lung cancer (7, 8). At the same time, long-term household use of clean energy mitigates the risk of climate extremes, improves outdoor living conditions and reduces the production and spread of disease (9). The use of clean energy increases the efficiency of tasks such as cooking and heating (10), saves time for residents to engage in productive activities, increases household income, and enhances disease prevention and treatment (11). The long-term use of clean energy in households significantly increases residents' life satisfaction and wellbeing (12, 13), thereby improving their mental health (14). The health effects of household clean energy consumption were more pronounced for women (15), particularly in terms of lower rates of maternal morbidity and mortality (16). In addition, the positive health effects of cleaner energy use are more pronounced in developing countries, with households using clean energy sources for instance LPG having higher levels of health than those using non-clean energy sources like as coal in Pakistan (17). In the case of China, Liu et al. (18) found that using clean energy reduced the odds of residents being diagnosed with chronic lung disease and heart disease in China's families. Likewise, Zhang et al. (19) analysis research data from China, and the findings revealed that household energy cleanliness improved the physical health of rural residents and improved the mental health of urban residents.

**Figure 1:**
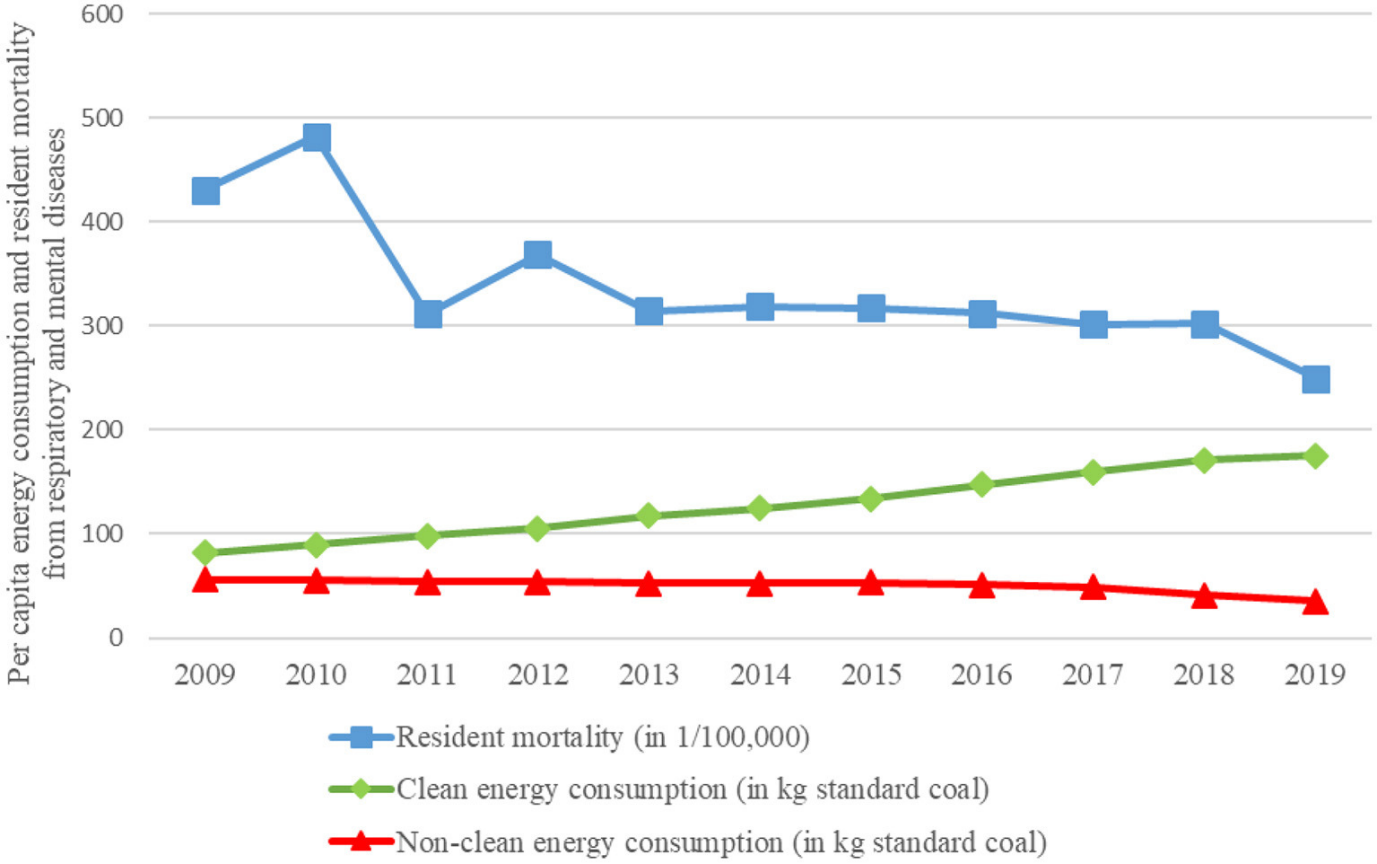
Per capita energy consumption and resident mortality from respiratory and mental diseases of China. Data source: China National Statistical Yearbook (2010–2020). In this paper, the units of different energy sources are uniformly converted into kilograms of standard coal according to the energy calorie conversion formula.

According to the current literature, long-term household use of clean energy is beneficial to residents' health. The macro-statistics of China support this viewpoint. This paper compiled and plotted data from China's National Statistical Yearbook on per capita energy consumption and resident mortality (respiratory disease mortality + mental disease mortality) from 2009 to 2019 (see Figure 1). As shown in Figure 1, per capita consumption of clean energy (electricity + LPG + natural gas) has been increasing, while consumption of non-clean energy (coal + coal gas) has been decreasing, indicating that China's household energy consumption is shifting to a cleaner energy. At the same time, residents' mortality rates from respiratory and mental diseases were declined. The choice of respiratory and mental disease mortality is based on existing research that suggests these two diseases are linked to household energy consumption (8, 20). As a result of Figure 1, it can be concluded that household clean energy consumption benefits residents' health. However, macro-statistics have limitations, which lack of information on household fuel use such as firewood, hay, cow dung, biogas, and solar energy. This problem can be addressed more effectively using micro-survey data. As a result, this paper examines the impact of household clean energy consumption on health using data from the 2018 China Health and Retirement Longitudinal Study (CHARLS).

A relatively consistent conclusion in the existing literature is that long-term use of clean energy can improve residents' health. But most studies have only reached this conclusion based on data analysis (5, 8), and few literature explored the internal mechanism of the impact of clean energy consumption on health based on theory (21). Meanwhile, some studies have used the Order Probit Model to analyze the relationship between energy consumption and health in empirical analysis, but few studies have dealt with potential endogenous problems (20). Furthermore, the current literature only discusses the impact of clean energy consumption on total health (9, 14), and does not analyze whether or how clean energy consumption impacts common diseases.

In summary, this paper makes five marginal contributions to the literature. First, the 2018 CHARLS data is used as a sample in this paper to provide new micro-evidence for the study of clean energy consumption and health. CHARLS focuses on collecting health data from Chinese residents, and using this data as a sample to study the health problems of micro-subjects would be more reliable (22). Second, this paper introduces a new theoretical analysis concept, and health and energy economics theory can fully reveal the impact of clean energy consumption on health. Third, to address the existing endogeneity problem, this paper employs the instrumental variable method and the conditional mixed process estimation method, which increases the credibility of this paper. Fourth, this paper discusses the heterogeneity of the health effects of clean energy consumption across genders, household economic conditions, and religious beliefs, adding to the findings of previous research. Fifth, this paper examines the impact of clean energy consumption on eight different common diseases, offering a fresh perspective for future research on the subject.

The remaining sections include, Theoretical analysis (Section 2); Data and method (Section 3); Empirical analysis (Section 4); Mechanism analysis (Section 5); Further research (Section 6); Conclusion and policy recommendations (Section 7).

## Theoretical analysis

Mushkin (23) identified health as a component of human capital and previously examined health issues from an economic standpoint. The classic paper by Arrow (24), “Uncertainty and Welfare Economics,” marked the establishment of health economics. Human capital theory and welfare economic theory have both become important theoretical foundations of health economics (25). Furthermore, Groosman (26) put forward the concept of health demand, believed that health can be regarded as an investment activity of people, and first proposed the health production function:


(1) $$\begin{array}{l} H=f\left(M,\;LS,\;E,\;S,X\right) \end{array}$$

The *H* represents health; *M* indicates healthcare; *LS* shows lifestyle; *E* stands for environment; *S* signifies schooling; and *X* shows other factors that affect health.

Some research enhanced the HPF and examined the dynamic interactions between various factors and health, with household economic condition, human capital (schooling), environment, society, and lifestyle serving as common HPF vectors (27, 28). Despite the fact that there have been few studies that incorporate energy (fuel) as a vector in the HPF, earlier research has demonstrated that household energy use is an important factor in health (29). Consequently, this study establishes the Household Health Production Function (HHPF) with energy consumption:


(2) $$\begin{array}{l} H=f\left(EC,\;W,HC,ES,EN,SC,X\right) \end{array}$$

The *H* indicates health; *EC* shows household energy consumption; *W* represent welfare; *HC* stands for human capital; *ES* signifies the household economic status; *EN* represents environment; *SC* is social contact; and *X* shows other important factors (i.e., age, gender, and etc.).

Energy is a basic requirement for household production and daily life. Household energy consumption, according to energy economics theory, is a decision process that seeks to maximize utility (30). To meet their utility needs, most households use multiple types of energy at the same time (21). In general, households use four types of energy: first, all clean energy, second, all non-clean energy, third, a mixture of clean and non-clean energy with a greater proportion of clean energy than non-clean energy, and fourth a mixture of clean and non-clean energy with a smaller proportion of clean energy than non-clean energy. To achieve Pareto dynamic optimization of energy consumption, households dynamically adjust their energy mix in response to changes in utility pursuits.

It is assumed that households choose the first energy consumption mix, using clean energy sources in various activities such as cooking and heating. Then clean energy does not produce harmful substances during the combustion process and does not pollute the indoor air, thus not harming health. At the same time, clean energy is more efficient than non-clean energy, saving time for productive, social contact, and educational activities for households, potentially leading to higher household income, increased economic wellbeing, and the accumulation of social and human capital, which in turn contributes to better health. Further, it is assumed that the household chooses the second energy consumption mix. Substances such as carbon monoxide generated during the combustion of non-clean energy will directly damage human health through the respiratory system. Meanwhile, the use of non-clean energy will also cause problems such as air pollution and environmental damage, which indirectly affect health.

In addition, it is assumed that household *A* chooses the third energy consumption mix and household *B* chooses the fourth energy consumption mix. Non-clean energy sources will then have a negative impact on the health of both families. However, because the proportion and frequency with which household *A* uses clean energy is greater than that of household *B*, household A's health level will be greater than that of household *B*. In summary, household energy use is included as a vector in the health production function in this paper. The analysis revealed that if households rely on non-clean energy excessively, they not only have a direct negative impact on health but also damage it indirectly through other pathways (e.g., air pollution, decreased wellbeing, etc.), whereas household clean energy consumption benefits residents' health.

**Figure 2:**
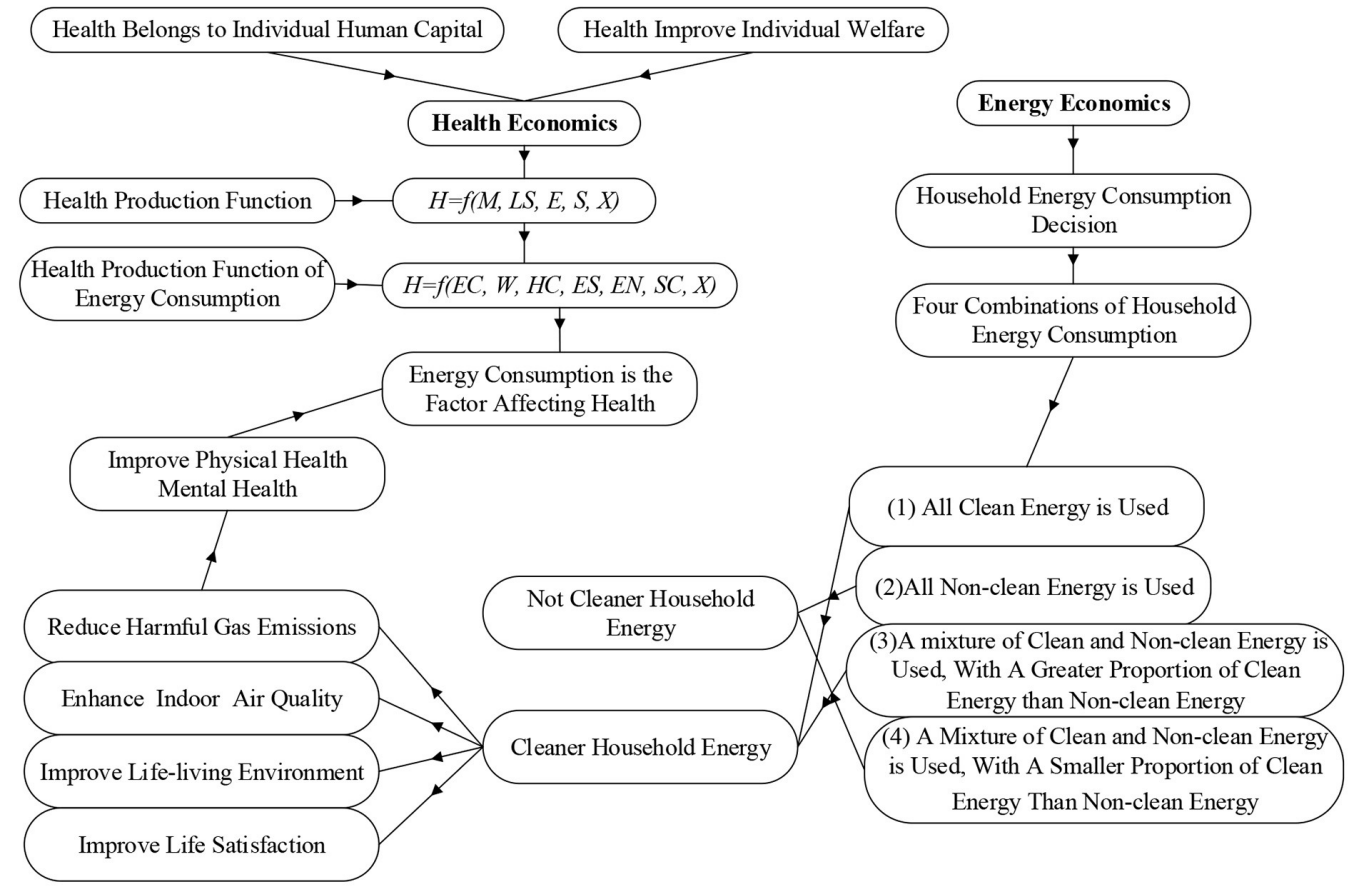
The theoretical mechanism of household clean energy consumption affecting health. Source: The author draws according to the content.

Figure 2 shows the theoretical analysis process of the impact of household clean energy consumption on health.

## Data and method

### Data

The sample for this study is the 2018 China Health and Retirement Longitudinal Study (CHARLS) data, which is published in September 2020. CHARLS is a longitudinal study that began in 2011 and provides definitive information on the health and aging of Chinese families (31). The 2018 questionnaire covers: demographic backgrounds; family information; health status and function; cognition and depression; informant information; health care and insurance; work and retirement; pension; income, expenditure, and assets; house property, and housing characteristics. The CHARLS covered 28 provinces of China, 150 countries/districts, and 450 villages/urban communities, which are representative at a national level. This paper first matches the data of each module according to the respondent ID; Then extract the data for variables related to this study from the data set; Next, the missing values of variables are processed by means of mean filling, median filling, and deletion, and the data is standardized by the Z-score method; Finally, 11,635 sample data were obtained for empirical analysis.

### Variables

#### Explained variable

Health. In the current literature on health issues from the perspective of microeconomics, “health self-assessment” is often used as a proxy variable for “health” (32). Because “health self-assessment” can reflect both the physical and mental health of micro-subjects to a certain extent (20). Therefore, we use residents' subjective health evaluation perceptions as a proxy variable for health, and the data of the survey question “What do you think about your health?” was chosen to measure resident's health.

#### Explanatory variable

Clean Energy Consumption (CEC). “Whether to use clean energy,” “the percentage of clean energy usage,” and “the frequency of clean energy usage” are three commonly used indicators to measure clean energy consumption (33). However, we cannot obtain data from CHARLS to calculate “the percentage of clean energy usage” and “the frequency of clean energy usage.” Therefore, according to the options in the questionnaire “what is the main source of cooking fuel?” this paper set to CEC = 1 if the respondent chooses clean energy (natural-gas, marsh-gas, LPG, or electricity) and CEC =0 if the respondent chooses non-clean energy (coal, crop-residue, or wood-burning).

#### Control variables

The individual characteristics, family economic status, and daily life situation of micro-subjects may all have an impact on their health (18). In empirical analysis, these factors are usually added to the model as control variables, so as to improve the accuracy of the regression results of the core explanatory variables and the explained variables. Therefore, this paper selects age, education level, income, and housing structure, etc. as the explained variables (34).

Table 1 presents the main variables of this paper.

**Table 1:** Variables selection and definition.

**Type**	**Variables**	**Definition**
Explained variables	Self-health evaluation (Health)	What do you think of your health? 1 = very poor; 2 = poor; 3 = fair; 4 = good; 5 = very good.
Explanatory variable	Clean energy consumption (CEC)	What is the main source of cooking fuel? Natural-gas, marsh-gas, liquefied-petroleum-gas and electric = clean energy = CEC = 1; coal, crop-residue, and wood-burning = non-clean energy = CEC = 0.
Control variables	Age	2018-Year of birth.
	Education	What's the highest level of education you have now (not including adult education)? 1 = illiterate; 2 = did not finish primary school, home school or elementary school; 3 = middle school, high school, vocational school, or associate degree; 4 = bachelor's degree, master's degree, or doctoral degree.
	Marriage	What is your marital status? 0 = never married; 1 = married; 2 = widowed, divorced and separated (don't live together as a couple anymore).
	Income	*Ln* (annual income) = *Ln* (wage income + business income + transfer income + property income + 1)
	Expenditure	*Ln* (annual expenditure) = *Ln* [(monthly expenditure) × 12 + 1]
	Debt	*Ln* (bank loan debt + credit card debt+ other debt)
	Insurance	Have you bought medical insurance? (Include public medial insurance and private commercial medical insurance), 1 = yes; 0 = no.
	Building structure (BS)	What type of structure is this building? 1 = Stone; 2 = Mongolian yurt/Woolen felt/Tent; 3 = Cave dwelling; 4 = Wood/Thatched; 5 = Adobe; 6 = Concrete and steel/Bricks and wood.
Instrumental variable	District	Respondent's residential district? 1 = rural; 2 = urban-rural combination; 3 = urban.
Heterogeneity test variables	Gender	1 = male; 0 = female.
	Poverty	From 2013 to 2018, was your family identified as a poor household by the government? 1=yes; 0=no.
	Religious	Are you religious? 1 = yes; 0 = no.
Mediating variable	Air quality (AQ)	Are you satisfied with the current air quality? 1 = not at all satisfied; 2 = not very satisfied; 3 = somewhat satisfied; 4 = very satisfied; 5 = completely satisfied.
	Happiness	Are you satisfied with your current life? 1 = not at all satisfied; 2 = not very satisfied; 3 = somewhat satisfied; 4 = very satisfied, 5 = completely satisfied.

In this paper, the mean, standard deviation, and maximum and minimum values of the main variables were calculated using Stata v15.0 software, and the calculation results are reported in Table 2. The mean of health is 3.13, indicating that residents are in good health, but still about 24% of respondents are unhealthy. There are 7,872 (67.66%) households using clean energy, and another 1/3 of the households are still using non-clean energy. Most of the respondents were middle-aged (mean age = 50.26), with a total of 6,965 (59.86%) residents between the ages of 41 and 60. The mean of education is 2.23, indicating that most of the residents have a low level of school education, and only 2.01% of the residents have received university education. 178 (1.53%) residents were not yet married, and among the 11,457 residents who were married, 17.82% were in an abnormal state of marriage (widowed, divorced, and separated). The income of the interviewed families was greater than the expenditure, and the gap between household income and expenditure was large, but most of the families were debt free. About 4/5 of the surveyed households purchased public health insurance. 69.15% of the household housing structure is concrete, steel, bricks, and wood. 6,598 (56.71%) of the surveyed households lived in rural areas. From 2013 to 2018, 25.10% of the surveyed households were identified as poor households by the Chinese government. More than half (55.95%) of the respondents were male. Most (72.51%) respondents do not believe in religion. About 10% of respondents are dissatisfied with their current life. Air quality has been significantly improved, and 85.80% of the respondents are satisfied with the current air quality.

**Table 2:** Descriptive statistics of the studied variables.

**Variables**	**Observations**	**Percentage**	**Mean**	**Std. dev**.	**Min**	**Max**
Health	11,635	100.00%	3.13	1.02	1	5
Health = 1	645	5.54%				
Health = 2	2,155	18.52%				
Health = 3	5,197	44.67%				
Health = 4	2,319	19.93%				
Health = 5	1,319	11.34%				
CEC	11,635	100.00%	0.76	0.43	0	1
CEC = 1	7,872	67.66%				
CEC = 0	3,763	32.34%				
Age	11,635	100.00%	51.26	9.63	18	97
18 ≤ Age ≤ 40	934	8.03%				
41 ≤ Age ≤ 60	6,965	59.86%				
61 ≤ Age ≤ 97	3,736	32.11%				
Education	11,635	100.00%	2.23	0.75	1	4
Education = 1	2,019	17.35%				
Education = 2	5,178	44.50%				
Education = 3	4,204	36.13%				
Education = 4	234	2.01%				
Marriage	11,635	100.00%	1.17	0.39	0	2
Marriage = 0	178	1.53%				
Marriage = 1	9,384	80.65%				
Marriage = 2	2,073	17.82%				
Income	11,635	100.00%	9.14	2.38	0.00	17.48
Expenditure	11,635	100.00%	8.96	1.61	0.00	14.51
Debt	11,635	100.00%	1.28	3.45	0.00	15.43
Insurance	11,635	100.00%	0.79	0.41	0	1
Insurance = 1	9,132	78.49%				
Insurance = 0	2,503	21.51%				
BS	11,635	100.00%	5.63	0.8	1	6
BS = 1	206	1.77%				
BS = 2	109	0.94%				
BS = 3	167	1.44%				
BS = 4	135	1.16%				
BS = 5	2,972	25.54%				
BS = 6	8,046	69.15%				
District	11,635	100.00%	1.61	0.77	1	3
District = 1	6,598	56.71%				
District = 2	2,964	25.47%				
District = 3	2,073	17.82%				
Gender	11,635	100.00%	0.56	0.5	0	1
Gender = 1	6,510	55.95%				
Gender = 0	5,125	44.05%				
Poverty	11,635	100.00%	0.25	0.43	0	1
Poverty = 1	2,920	25.10%				
Poverty = 0	8,715	74.90%				
Religious	11,635	100.00%	0.28	0.45	0	1
Religious = 1	3,198	27.49%				
Religious = 0	8,437	72.51%				
AQ	11,635	100.00%	3.29	0.83	1	5
AQ = 1	329	2.83%				
AQ = 2	1,323	11.37%				
AQ = 3	5,089	43.74%				
AQ = 4	4,420	37.99%				
AQ = 5	474	4.07%				
Happiness	11,635	100.00%	3.36	0.80	1	5
Happiness = 1	327	2.81%				
Happiness = 2	857	7.37%				
Happiness = 3	5,258	45.19%				
Happiness = 4	4,689	40.30%				
Happiness = 5	504	4.33%				

### Econometric model

As “Health” is an ordered multi-category variable, valid estimates may not be obtained if OLS and bivariate Probit models are used. The ordered probit (O-probit) model can meet the requirements of the data structure (5), and Greene et al. (35) uses the ordered probit model to explore the question of health in Australia. Therefore, the main model in this paper is:


(3) $$\begin{array}{l} Health_{i}^{*}=\omega_{n}+\beta_{n}\times CEC+\varphi_{n}\times CV_{r}+\mu_{k} \end{array}$$

(4) $$Health^{*}=\{\begin{array}{c} i=1\\ i=2\\ i=3\\ i=4\\ i=5 \end{array}$$

The $Health_{i}^{*}$ is the latent variable for health; *i* = 1, 2, 3, 4, 5 denotes five self-evaluations of health; ω_*n*_ is the intercept term, β_*n*_ and φ_*n*_ are regression coefficients; *CEC* is clean energy consumption. *CV*_*r*_ is the control variables. μ_*k*_ denotes the error term.

To examine the mediating and moderating effects of clean energy consumption and health, this paper refers to Wen et al. (36) approach and set up a mediating effects model as:


(5) $$\{\begin{array}{c} Health_{i}^{*}=\omega_{n}+\beta_{n}\times CEC+\varphi_{n}\times CV_{r}+\mu_{k}\;\\ MV=\omega_{1}+\beta_{1}\times CEC+\varphi_{1}\times CV_{r}+\mu_{1}\;\\ Health_{i}^{*}=\omega_{2}+\beta_{2}\times CEC+\rho \times MV+\varphi_{2}\times CV_{r}+\mu_{2} \end{array}$$

Where *MV* is the mediating variable, and ρ is the regression coefficient of the mediating variable. If β_*n*_,β_1_,β_2_ and ρ are all significant, it means that *MV* has a mediating effect on *CEC* and $Health_{i}^{*}$.

## Empirical analysis and discussion

### Basic regression

We consider that if there is a multi-collinearity issue among variables, it will lead to serious deviations in the regression results. Therefore, the multi-collinearity test was carried out in this study before the regression. The variance inflation factor (VIF) is a common indicator to measure multi-collinearity. The VIF of this paper is 5.63 <10, which means that there is no multi-collinearity issue between the variables selected in this paper (37).

The results from models (1) show that clean energy consumption is significantly and positively associated with health, indicating that the use of clean energy by households can improve the health of residents (38). The trend in the average marginal effect values in the results of model (2) shows that the use of clean energy can gradually improve the health of the residents.

*Age* is negatively correlated with health under the significance standard of 0.01. With the increase of age, the functions of human organs and the immune system decline, and they are prone to diseases (39). Furthermore, at the 0.01 level of significance, *education* is positively associated with health, as higher education is associated with higher returns on educational investment, better jobs, income levels, and a greater ability to prevent and treat disease (40). Likewise, this study also revealed a significant positive correlation between *income* and health. The greater the willingness and ability of residents to invest in health, the greater their willingness and ability (41). *Expenditure* is significantly and negatively correlated with health, as the more items and amounts a household spends, the less it must spend on savings and investments, the less it is able to invest in health and fight disease, and the more it is vulnerable to health risks (42). In addition, medical *insurance* is significantly and positively correlated with health, and medical insurance has the function of defusing and hedging health risk (43). *Building structure* is positively correlated with health, firstly because a safer housing structure indicates a higher level of household income and the ability to cope with disease crises (44), and secondly because households with a safe housing structure can withstand the risks to human health caused by climatic disasters and environmental degradation (45).

As shown in Table 3, *marriage* is not related to health, which is different from the conclusions of some current studies (46). It is observed that the regression coefficient of marriage is 0.014 > 0, indicating that marriage will have a positive effect on health (47). *Debt* is not related to health, which is different from the conclusions of Clayton et al. (48) and Andelic and Feeney (49), which may be related to the sample data in this paper and the debt structure of Chinese residents.

**Table 3:** The regression results of CEC and health.

	**O-probit (1)**	**O-probit (2) (marginal effect)**				
**Variables**	**Health**	**Health = 1**	**Health = 2**	**Health = 3**	**Health = 4**	**Health = 5**
CEC	0.054^**^ (0.025)	−0.006^**^ (0.003)	−0.011^**^ (0.005)	−0.002^**^ (0.001)	0.009^**^ (0.004)	0.010^**^ (0.005)
Age	−0.004^***^ (0.001)					
Education	0.016^***^ (0.004)					
Marriage	0.014 (0.027)					
Income	0.034^***^ (0.004)					
Expenditure	−0.014^***^ (0.006)					
Debt	0.003 (0.002)					
Insurance	0.056^**^ (0.027)					
BS	0.020^**^ (0.010)					
Observations	11,635	11,635	11,635	11,635	11,635	11,635

Robust standard errors in parentheses

*** p < 0.01,

** p < 0.05. CEC, clean energy consumption; 1, clean energy; 0, non-clean energy; Health, self-health evaluation; 1, very poor; 2, poor; 3, fair; 4, good; 5, very good; Insurance, medical insurance; BS, Building structure.

### Robustness test

This paper uses three approaches for robustness tests, and the results of the robustness tests are reported in Table 4. First, replace the O-probit model with an ordered logit (O-logit) model (Model 1). Second, the sample size was reduced: the life expectancy per capita in China was 77 years in 2018 (50). Because CHARLS primarily collected health data from people aged 45 and up, samples younger than 45 and older than 77 years were excluded and then regressed (Model 2). Third, the 2018 CHARLS sample set was replaced by the 2018 China Family Panel Studies (CFPS) and the 2018 Chinese General Social Survey (CGSS). CFPS is a nationwide, comprehensive social tracking survey designed to reflect social, economic, demographic, educational, and health changes in China by tracking and collecting data at the individual, household, and community levels (51). CGSS is the earliest national, comprehensive, and continuous academic survey project in China that systematically and comprehensively collects data at multiple levels of society, communities, households, and individuals (52). We extract data from CFPS and CGSS for the same metrics as in this paper; define and calculate “Health,” “CEC,” and control variables in the same way as in this paper; and use the same model (O-probit) to analyze the relationship between clean energy consumption and residents' health (Model 3 and 4).

**Table 4:** The results of robustness test of CEC and health.

	**Replace model**	**Reduce sample**	**Replace sample set**	
	**O-logit (1)**	**O-probit (2)**	**O-probit (3) CFPS_2018**	**O-probit (4) CGSS_2018**
**Variables**	**Health**	**Health**	**Health**	**Health**
CEC	0.090^**^ (0.043)	0.062^**^ (0.030)	0.072^***^ (0.018)	0.146^***^ (0.019)
CV	Control	Control	Control	Control
Observations	11,635	10,666	13,502	12,781

Robust standard errors in parentheses

*** p < 0.01,

** p < 0.05. CEC, clean energy consumption; Health, self-health evaluation; CV, control variables.

As it can be seen in Table 4, clean energy consumption was significantly positively associated with health after robustness tests using three different approaches. The robustness test results support the findings of the basic regression, indicating that the analysis results in this paper are reliable, that is, the long-term use of clean energy in households can significantly improve the health of residents.

### Endogenous discussion and treatment

We cannot add all the factors that affect residents' health as control variables to the model for regression, and there may be errors between residents' self-health evaluation and their real health status. This paper may have endogenous issues caused by “missing variables” and “self-selection bias,” resulting in errors in regression coefficients. In this paper, “respondent's residential district (District, 1 = rural, 2 = urban-rural combination, 3 = urban)” was selected as the instrumental variable (IV), and the Iv-O-probit models were used to deal with possible endogenous issues. IV must meet two basic requirements: first is correlation (IV are related to endogenous variables); and second is exclusivity (IV are not related to control variables, explained variables, and error terms). “District” meets the correlation requirements since households living in different districts have different energy consumption due to differences in energy resource endowments (53, 54), thus “District” is related to “CEC.” Some literature believes that rural residents are healthier than urban residents, because of rural residents have a green lifestyle (55). Other studies have found that the health level of urban residents is higher than that of rural residents (56), which may be because cities have more convenient medical resources so as to get more health care. This means that there is no strict causal relationship between “District” and “Health” (57). Therefore, “District” conforms to exclusivity, and it is reasonable to use “District” as an IV in this paper.

The explained variable health in this paper is an ordered multi-category variable, and it is still technically difficult to directly use the IV in combination with O-probit. Therefore, in this paper, we refer to Roodman (58) and use a combination of instrumental variables approach and conditional mixed process (CMP) estimation to deal with the endogenous of the O-probit model. Table 5 reports the results of the Iv-O-probit model for the endogenous problem.

**Table 5:** The results of endogenous treatment of CEC and health with CMP estimation method.

	**First stage**		**CMP estimation method**					
	**O-probit (1)**	**Probit (2)**	**Iv-O-probit (3)**	**Iv-O-probit (4)** **(Marginal effect)**				
**Variables**	**Health**	**CEC**	**Health**	**Health = 1**	**Health = 2**	**Health = 3**	**Health = 4**	**Health = 5**
CEC	0.054^**^ (0.025)		0.072^**^ (0.033)	−0.005^**^ (0.002)	−0.010^**^ (0.004)	−0.002^**^ (0.001)	0.009^**^ (0.004)	0.019^**^ (0.009)
District	0.012 (0.010)	0.096^***^ (0.026)						
atanhrho_12(P)			0.000	0.000	0.000	0.000	0.000	0.000
F statistics	242.4							
CV	Control	Control	Control	Control	Control	Control	Control	Control
Observations	11,635	11,635	11,635	11,635	11,635	11,635	11,635	11,635

Robust standard errors in parentheses

*** p < 0.01,

** p < 0.05. CEC, clean energy consumption; Health, self-health evaluation; District, the area where the respondents lived; 1, rural; 2, urban-rural combination; 3, urban; CV, control variables.

In Table 5, the results of models (1) and (2) show that the IV (District) is significantly correlated with the explanatory variable “CEC” and not with the explanatory variable “Health,” which statistically meets the requirements of IV. The auxiliary estimation parameter atanhrho_12 is significantly different from 0 (*P* = 0), indicating that there is a significant correlation between the two equations in the joint cubic equation model and that adopting the CMP estimation method is more effective than estimating them separately, also demonstrating that “CEC” is an endogenous variable. The results of model (3) indicate that “CEC” is significantly and positively associated with “Health” after instrumental variables approach with CMP estimation. Compared to the basic regression, the coefficient of 0.072 > 0.054 and the increased average marginal effect value at each cut-off point indicate that the positive health effects of clean energy consumption are underestimated in the base regression. The first stage F-statistic value of 242.4 is greater than the experiential value of 10, indicating that there is no weak instrumental variable problem.

### Heterogeneity analysis

In China, women carry out more work within the home than men, and use energy more frequently than men. Twumasi et al. (5) found that the risks to women's health from using non-clean energy were more significant. The results of model (1) in Table 6 show that clean energy consumption is positively associated with men's and women's health at the 0.05 level of significance, and the regression coefficient (0.071 > 0.039) shows that household clean energy consumption has a stronger effect on improving women's health.

**Table 6:** The results of heterogeneity analysis of CEC and health.

	**O-probit (1)**		**O-probit (2)**		**O-probit (3)**	
	**Male**	**Female**	**Poverty = 1**	**Poverty = 0**	**Religious = 1**	**Religious = 0**
**Variables**	**Health**	**Health**	**Health**	**Health**	**Health**	**Health**
CEC	0.039^**^ (0.017)	0.071^**^ (0.034)	0.137^**^ (0.058)	0.058^**^ (0.025)	0.015 (0.067)	0.074^***^ (0.025)
CV	Control	Control	Control	Control	Control	Control
Observations	6,510	5,125	2,920	8,715	3,198	8,437

Robust standard errors in parentheses

*** p < 0.01,

** p < 0.05. CEC, clean energy consumption; Health, self-health evaluation; Poverty, poor family; 1, yes; 0, no; Religious, religious brief; 1, yes; 0, no; CV, control variables.

The economic status of the household is directly influenced by energy choices. According to the poverty theory of development economics, economically poor households are also more likely to be energy poverty and have a higher reliance on non-clean energy sources (33). The results of model (2) in Table 6 show that clean energy consumption is positively associated with health regardless of whether the household is in poverty or not, but the coefficient values show that clean energy consumption has a more obvious effect on improving the health of poor households.

Religious households regularly incur expenditure on religious activities, have less money to spend on clean energy, and are more likely to use non-clean energy. Simultaneously, some religious teachings may discourage residents from utilizing clean energy (59). The results of model (3) in Table 6 show that clean energy consumption is positively associated with the health of residents who are not religious and not associated with the health of residents who are religious.

## Mechanism analysis: Mediating effect test

The use of clean energy in the home increases the life satisfaction (happiness) of residents (60, 61). Residents with high life satisfaction are more concerned about health and less likely to suffer from mental illness. The results of models (1), (2), and (3) in Table 7 show that clean energy consumption increases resident happiness at a significance criterion of 0.01 and is thus significantly and positively associated with residents' health. The corresponding *p-*values of the Soble and Bootstrap tests are both <0.05, indicating that happiness plays a partial mediating role in clean energy consumption impact on health.

**Table 7:** The results of mediating effect of CEC, happiness and AQ on health.

	**O-probit (1)**	**O-probit (2)**	**O-probit (3)**	**O-probit (4)**	**O-probit (5)**
**Variables**	**Health**	**Happiness**	**Health**	**AQ**	**Health**
CEC	0.054^**^ (0.025)	0.082^***^ (0.024)	0.075^***^ (0.023)	0.085^***^ (0.023)	0.075^***^ (0.023)
Happiness			0.0274^**^ (0.0123)		
AQ					0.030^**^ (0.012)
Soble test (*p*)		0.046 < 0.05		0.036 < 0.05	
Bootstrap (500)		Direct effect (*p* = 0.058 < 0.10)		Direct effect (*p* = 0.025 < 0.05)	
		Indirect effect (*p* = 0.001 < 0.01)		Indirect effect (*p* = 0.001 < 0.01)	
CV	Control	Control	Control	Control	Control
Observations	11,635	11,635	11,635	11,635	11,635

Robust standard errors in parentheses

*** p < 0.01,

** p < 0.05. CEC, clean energy consumption; Health, self-health evaluation; Happiness, self-life satisfaction; 1, not at all satisfied; 2, not very satisfied; 3, somewhat satisfied; 4, very satisfied; 5, completely satisfied; AQ, air quality satisfaction; 1, not at all satisfied; 2, not very satisfied; 3, somewhat satisfied; 4, very satisfied; 5, completely satisfied; CV, control variables.

Household use of non-clean energy pollutes the air and reduces indoor air quality (AQ) (62). Harmful products of energy combustion enter the body through human respiration, causing harm to the health of residents. This paper uses residents' subjective evaluation of air quality as a proxy variable for air quality and conducts a mediating effects analysis. The results of models (1), (4), and (5) in Table 7 show that the long-term use of clean energy significantly enhances air quality and thus improves the health of the residents. The *p*-values for the corresponding Soble and Bootstrap tests were <0.05, indicating that air quality plays a partially mediating role in clean energy consumption and health.

## Further research: Nexus between CEC and eight different common diseases

Chronic diseases have become a global health concern. Obesity, hypertension, hyperlipidemia, diabetes, cancer, lung disease, stroke, asthma, osteoporosis, and kidney disease are the main chronic diseases with increasing diagnosis and mortality rates in the world (3). Deaths from chronic diseases accounted for 88.5% of deaths in China in 2019, with 80.7% of deaths from cardiovascular diseases, cancer, and chronic respiratory diseases (50). Households that used non-clean energy sources were more likely to develop diseases such as cardiovascular disease and asthma (63). Therefore, this paper further discusses the impact of clean energy consumption on common chronic diseases.

It can be seen from Table 8, the results of model (1) show that clean energy consumption significantly reduces the prevalence of hypertension; the results of model (2) illustrate that clean energy consumption is negatively associated with hyperlipidemia at the 0.01 level of significance; the results of model (5) indicate that the long-term use of clean energy significantly suppresses the prevalence of lung disease; and the results of model (7) demonstrate that clean energy use is significantly negatively associated with asthma. The result of models (3), (4), and (6) indicated that the use of clean energy was negatively associated with diabetes, cancer, and stroke, respectively.

**Table 8:** The regression results of CEC and eight different common diseases.

	**Probit (1)**	**Probit (2)**	**Probit (3)**	**Probit (4)**	**Probit (5)**	**Probit (6)**	**Probit (7)**	**OLS (8)**
**Variables**	**Hypertension**	**Hyperlipidemia**	**Diabetes**	**Cancer**	**Lung**	**Stroke**	**Asthma**	**Depression**
CEC	−0.092^***^ (0.028)	−0.038^***^ (0.003)	−0.046 (0.040)	−0.072 (0.076)	−0.134^***^ (0.032)	−0.052^***^ (0.038)	−0.088^***^ (0.030)	−0.025^***^ (0.008)
CV	Control	Control	Control	Control	Control	Control	Control	Control
Constant	−0.292^***^ (0.024)	−0.747^***^ (0.026)	−1.638^***^ (0.040)	−2.241^***^ (0.065)	−1.014^***^ (0.029)	−1.415^***^ (0.035)	−1.524^***^ (0.037)	0.606^***^ (0.007)
Observations	11,635	11,635	11,635	11,635	11,635	11,635	11,635	11,635

Robust standard errors in parentheses

*** p < 0.01. CEC, clean energy consumption; Different diseases, are you diagnosed with Hypertension/Hyperlipidemia/Diabetes/Cancer/Lung disease/Stroke/Asthma? 1, yes; 0, no; Depression, depression index calculates by the factor analysis model; CV, control variables.

In recent years, depression has become a serious health issue that has plagued society (64). Long-term use of non-clean energy can lead to psychological and mental illness (19). This paper refers to Zhang et al. (65) and select data from seven research questions and take the factor analysis method to measure the depression index as a proxy variable for depression. The seven questions including: *(1) I had trouble keeping my mind on what I was doing; (2) I felt depressed; (3) I felt everything I did was an effort; (4) I felt hopeful about the future; (5) I felt fearful; (6) I was happy; (7) I felt lonely.”* The answer to each question is “*1* = *rarely or none of the time, 2* = *some or a little of the time, 3* = *occasionally or a moderate amount of the time, 4* = *most or all of the time.”* In Table 8, the results of model (8) show that the use of clean energy significantly reduces the probability of diagnosed depression among residents.

## Conclusion and policy recommendations

### Conclusions

Recently, both developing and developed countries around the world have committed to using cleaner energy and addressing health issues. Based on health economics and energy economics theory, this paper first examines the impact mechanism of household energy consumption on residents' health. The data from the 2018 CHARLS is used as a sample in an econometric model to investigate whether and how clean energy consumption affects residents' health. This study discovered that long-term use of clean energy can significantly improve residents' health. Simultaneously, household clean energy consumption has a greater impact on the health of women, low-income households, and non-religious residents. Furthermore, the mechanism analysis revealed that subjective happiness and air quality play a partial role in mediating the impact of energy consumption on residents' health. Furthermore, long-term use of clean energy reduced the incidence of hypertension, hyperlipidemia, lung disease, asthma, and depression.

### Discussion

Using both theoretical and empirical analyses, this paper verifies the positive impact of clean energy consumption on health, similar to the findings of Twumasi et al. (5), Liao et al. (7), and Wang et al. (16), etc., The contributions of this paper include: (1) using health economics and energy economics theories to analyze the underlying mechanisms of clean energy consumption affecting health; (2) not only analyzing whether clean energy consumption affects residents' health but also discussing how it affects health using mediating effect models; (3) not only analyzing the impact of clean energy consumption on overall health but also studying the relationship between clean energy and common chronic diseases and depression. Meanwhile, there are some limitations to this paper, such as the sample data is from China and the conclusions drawn may only be applicable to China or developing countries (regions) and are not of global relevance. Therefore, this paper provides ideas for further research: (1) Health economics and energy economics theories can be used to lay the groundwork for research on the impact of energy use on health; and (2) scholars can select data from different countries/regions (e.g., China and the United States, Europe and Africa, South Asia, and Western Europe, etc.) for repeated validation and comparative analysis.

### Policy recommendations

This study makes three policy recommendations in light of the conclusions.

*First*, the government first utilizes macro policies to modify the market pricing of clean energy and non-clean energy, reduce the household consumption expenses of clean energy, and boost the consumption demand for clean energy, thereby encouraging households to use clean energy for an extended period of time.

*Second*, the government provides financial incentives to households in urban areas to upgrade their fuel-energy infrastructure and to hasten the development of clean-burning stoves for those living in rural areas (especially poor households). Financial subsidies will be given to households implementing clean energy facilities to improve their clean energy consumption abilities.

*Third*, community and rural management organizations play the role of social education, publicize the effect of clean energy consumption, and increase residents' willingness to use clean energy. At the same time, community and rural management organizations should carry out health education activities to raise the health awareness of residents (especially female residents).

## Data availability statement

The raw data supporting the conclusions of this article will be made available by the authors, without undue reservation.

## Author contributions

Material preparation, data collection, and analysis were performed by FL and YD. The first draft of the manuscript was written by FL, YD, WL, DZ, and AC. All authors commented on previous versions of the manuscript, contributed to the study conception and design, and read and approved the final manuscript.

## Funding

This study was supported by the Youth Project of National Social Science Foundation of China (grant number 17CGL012) and the Key Project of Social Science Planning of Sichuan Province (grant number SC21A016).

## Conflict of interest

The authors declare that the research was conducted in the absence of any commercial or financial relationships that could be construed as a potential conflict of interest.

## Publisher's note

All claims expressed in this article are solely those of the authors and do not necessarily represent those of their affiliated organizations, or those of the publisher, the editors and the reviewers. Any product that may be evaluated in this article, or claim that may be made by its manufacturer, is not guaranteed or endorsed by the publisher.
